# The lipid linked oligosaccharide polymerase Wzy and its regulating co-polymerase, Wzz, from enterobacterial common antigen biosynthesis form a complex

**DOI:** 10.1098/rsob.220373

**Published:** 2023-03-22

**Authors:** Miriam Weckener, Laura S. Woodward, Bradley R. Clarke, Huanting Liu, Philip N. Ward, Audrey Le Bas, David Bhella, Chris Whitfield, James H. Naismith

**Affiliations:** ^1^ Structural Biology, The Rosalind Franklin Institute, Harwell Campus, Didcot OX11 0QS, UK; ^2^ Division of Structural Biology, University of Oxford, Roosevelt Drive, Oxford OX3 7BN, UK; ^3^ Centre Biomedical Sciences, North Haugh, University of St Andrews, St Andrews KY16 9ST, UK; ^4^ Department of Molecular and Cellular Biology, The University of Guelph, Guelph, ON, Canada; ^5^ MRC—University of Glasgow Centre for Virus Research, University of Glasgow, Sir Michael Stoker Building, Garscube Campus, 464 Bearsden Road, Glasgow G61 1Q, UK

**Keywords:** lipid, oligosaccharides, polymerase, Wzy, regulating

## Abstract

The enterobacterial common antigen (ECA) is a carbohydrate polymer that is associated with the cell envelope in the *Enterobacteriaceae*. ECA contains a repeating trisaccharide which is polymerized by WzyE, a member of the Wzy membrane protein polymerase superfamily. WzyE activity is regulated by a membrane protein polysaccharide co-polymerase, WzzE. Förster resonance energy transfer experiments demonstrate that WzyE and WzzE from *Pectobacterium atrosepticum* form a complex *in vivo*, and immunoblotting and cryo-electron microscopy (cryo-EM) analysis confirm a defined stoichiometry of approximately eight WzzE to one WzyE. Low-resolution cryo-EM reconstructions of the complex, aided by an antibody recognizing the C-terminus of WzyE, reveals WzyE sits in the central membrane lumen formed by the octameric arrangement of the transmembrane helices of WzzE. The pairing of Wzy and Wzz is found in polymerization systems for other bacterial polymers, including lipopolysaccharide O-antigens and capsular polysaccharides. The data provide new structural insight into a conserved mechanism for regulating polysaccharide chain length in bacteria.

## Graphical abstract

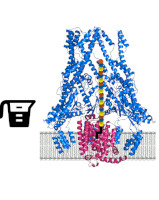

## 1. Introduction

Gram-negative bacteria are defined by a complex cell envelope format, which includes an outer membrane that provides a permeability barrier, which excludes large molecules including some antibiotics [1], affords structural strength [2] and helps protect bacteria from components of host immune defenses [3]. The inner leaflet of the outer membrane is formed of glycerophospholipids, but the outer leaflet consists almost entirely of a characteristic glycolipid called lipopolysaccharide (LPS). LPS molecules are complex structures [4], containing a highly conserved lipid A molecule made up of a phosphorylated disaccharide modified with four to seven acyl chains [5]. Lipid A is itself glycosylated by the core oligosaccharide (core OS), which shows some structural variation between species. In many bacteria, the core OS is further decorated by an O-antigen polysaccharide (OPS). The OPS polymer exhibits heterogeneous chain length distribution and repeat unit composition can vary considerably between species and between O-antigen serotypes within a species [3]; for example, there are more than 180 different O-antigens known for *Escherichia coli* [6]. Bacteria in which the OPS-biosynthesis genes have been deleted show reduced virulence due to impaired resistance to the immune response [3]. The diversity of OPS arises from the identities of the sugars that comprise the repeating unit, how they are connected, the extent of any branching to the linear arrangement, and chemical modification (e.g. acetylation) [3].

LPS is not the sole polysaccharide associated with the outer membrane or found on the cell surface of Gram-negative bacteria. Some bacteria are also protected from the immune system by a layer of high-molecular weight capsular polysaccharides (CPSs) [7]. Like OPS, CPSs have hypervariable repeat unit structures with more than 80 capsular serotypes in *E. coli*. Most members of the Enterobacteriaceae also produce a carbohydrate polymer called the enterobacterial common antigen (ECA) that is associated with the cell envelope. ECA can be linked to either diacylglycerolphosphate (ECA_PG_) or LPS lipid A core (ECA_LPS_), or it can exist in a lipid-free cyclic form in the periplasm (ECA_CYC_), and its precise role (or roles) in bacterial physiology is enigmatic [8]. ECA contains a trisaccharide repeat unit (*N*-acetyl-d-glucosamine-(α1–4)-*N*-acetyl-d-mannosaminuronic acid(β1–4)-4-acetamido-4,6-dideoxy-d-galactose) [8].

ECA [8], most OPSs [3] and some CPSs [7] are synthesized by a conserved strategy, employing an assembly line of enzymes often referred to as the Wzx/Wzy pathway [9]. In ECA biosynthesis, the enzymatic cascade is initialized by a phosphoglycosyltransferase called WecA, and this enzyme participates in production of OPSs in many bacteria, including all OPS types in *E. coli* [3,8]. WecA transfers an acetamido sugar phosphate onto membrane-embedded undecaprenyl phosphate (Und-P) at the interface with the cytosol. In other OPSs and many CPSs, bacteria use a homologue of the WbaP phosphoglycosyltransferase to transfer a hexose phosphate to Und-P [3,7]. In each case, the reaction products are elaborated into an Und-PP-linked oligosaccharide repeat unit by membrane-associated glycosyltransferases. The lipid-linked repeat unit is then translocated across the inner membrane into the periplasm by a flippase Wzx (designated WzxE in ECA to distinguish it from homologues from other examples of the assembly strategy found in the same cell). Wzx proteins belong to the multidrug and toxic compound extrusion (MATE) exporter family [10]. Once flipped, ECA repeat units are polymerized by transfer of the growing chain from its Und-PP carrier to the non-reducing terminus of an incoming Und-PP-linked repeat unit. This reaction is catalysed by the integral membrane polymerase WzyE, which belongs to a larger shape, elongation, division and sporulation (SEDS) glycosyltransferase family [11]. After polymerization, the fate of the final polysaccharide polymer depends on the pathway. OPS (and presumably ECA_LPS_) is transferred onto the lipid A core by the O-antigen ligase WaaL [3,12,13] and transferred to the outer leaflet of the outer membrane by the LPS transport (Lpt) machine [14]. Diacylglycerol phosphate is added to ECA_PG_ from a phosphatidylglycerol donor [15], but the enzyme responsible is uncertain, as is the export fate of this species. In Wzy-polymerized CPS, the polymer is transported to the surface through the dedicated outer membrane translocon Wza [16,17].

A characteristic feature of the Wzy-polymerization systems for OPS, ECA and CPS is the regulation of the polymerization phase by inner membrane proteins belonging to the polysaccharide co-polymerase (PCP) family [18]. This is best documented for OPS, where a PCP-1 protein (called Wzz) controls polymerization to create an abundant fraction of OPS species with a more homogeneous (modal) distribution of chain lengths [3]. A related PCP-1 protein (WzzE) operates in ECA biosynthesis [8,18]. Wzz proteins consist of two transmembrane helices flanking a large periplasmic domain; there is no cytoplasmic domain. The periplasmic domains of various Wzz homologues were found to crystallize in different oligomerization states [19–21] and the structures of full length, decameric WzzB_SE_ (from OPS biosynthesis in *Salmonella enterica* serovar Typhimurium) and octameric WzzB_EC_ (OPS biosynthesis in *E. coli* K-12) were recently solved by cryo-electron microscopy (cryo-EM) [22,23]. Wzz homologues have also been reported as (non-octameric) oligomers in a native-like lipid environment using cryo-EM [24]. All Wzz structures show a central cavity in the membrane region that is presumably filled by membrane lipids [22–24]. Wzy-dependent polymerization of CPS in Gram-negative bacteria exploits a more complicated PCP-2a protein (Wzc from *E. coli* is the prototype), distinguished from PCP-1 proteins by possession of an additional functionally essential cytoplasmic kinase domain [7]. Wzc is an octamer and the transmembrane helices are arranged in a non-close packed circular arrangement that creates a central cavity in the membrane similar to Wzz [25].

No direct catalytic activity has been described for any Wzz protein but mutants lacking *wzz* can no longer produce high-molecular weight modal OPS, suggesting it plays a regulatory and/or structural role [3]. The regulation of the OPS polymer length has been reconstituted *in vitro* by addition of Wzz to Wzy suggesting they may form a complex [26]. This concept is supported by *in vivo* cross-linking and co-immunoprecipitation studies in *Shigella flexneri* [27–30] and a yeast two hybrid experiment in *Rhizobium leguminarosum* [31]. The C-terminal transmembrane helix in Wzz appears to interact with Wzy and multiple residues in Wzy have been shown to be important for the interaction with Wzz [28–30]. Wzz has also been shown to co-immunoprecipitate with O-antigen, suggesting a direct interaction with the reaction product [28–30,32,33]. Mutations that affect OPS chain length are distributed throughout Wzz structures [34–36]. Notably variants of Wzz that are unable to oligomerize are unable to regulate chain length [36,37], but no simple relationship between polymer chain length and Wzz oligomerization state has been established [19]. This has led to several hypotheses for Wzz's mode of action [13,18,19,22,36,38–40], including: (i) the circumference of the periplasmic domain acting as some form of ruler for chain length [36]; (ii) Wzz as a scaffold around which multiple Wzy molecules assemble [19]; (iii) Wzz binding the growing OPS in such a way as to facilitate catalysis until polymer length breaks the interaction [40]; and (iv) OPS extending within the lumen of Wzz until it reaches the top of the periplasmic domain [13].

Here, we report a series of biochemical and structural studies on WzyE_PA_ and WzzE_PA_ from the ECA pathway of *Pectobacterium atrosepticum* (77% and 70% sequence identity to *E. coli* K12 WzyE and WzzE, respectively) that validate and characterize complex formation *in vitro* and *in vivo*. These findings support a general molecular model for how the polymerization of Wzy is regulated by Wzz.

## 2. Results

### 2.1. Isolation and validation of the WzyE_PA_/WzzE_PA_ complex

**Figure 1.**
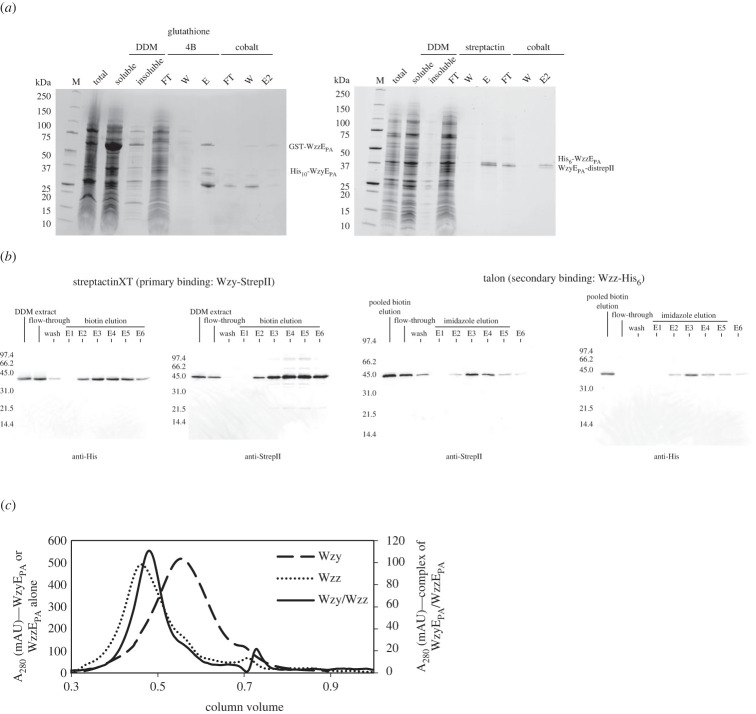
Isolation of Wzy : Wzz complexes. (*a*) SDS–PAGE of purifications of different combinations of affinity-tagged WzyE_PA_/WzzE_PA_ complexes. The left panel shows GST-WzzE_PA_ in complex with WzyE_PA_-His_10_ and was purified by first applying the protein to glutathione 4B resin and then applying the eluate to cobalt resin. Note that the GST fusion substantially changes the size of the corresponding Wzz fusion. The predicted molecular weights of the fusion proteins are 66 kDa for GST-WzzE_PA_, 55.5 kDa for WzyE_PA_-His_10_, 57 kDa for WzyE_PA_-distrepII and 42 kDa for His_6_-WzzE_PA_. M, marker; FT, flow-through; W, wash and E, eluate. E2 denotes the eluate from the second affinity column. The right panel shows the SDS–PAGE gel of the purification of the His_6_-WzzE_PA_/WzyE_PA_-distrepII complex by streptactin resin followed by cobalt resin. (*b*) Western blot images of samples taken during tandem affinity purification of His_6_-WzzE_PA_ and WzyE_PA_-distrepII by streptactin pulldown followed by TALON affinity chromatography. The western blots on the left are probing the samples taken after streptactin pulldown with the anti-His antibody recognizing His_6_-WzzE_PA_ and the anti-strepII antibody recognizing WzyE_PA_-distrepII. The western blots on the right show the samples taken during the cobalt affinity column, probed with the same antibodies as before. The predicted molecular weights of the fusion proteins are 57 kDa for WzyE_PA_-distrepII and 42 kDa for His_6_-WzzE_PA_. E1–6 denote eluate fractions 1–6, collected during elution. (*c*) The SEC profiles of individually expressed and purified WzyE_PA_-distrepII and His_6_-WzzE_PA_ and the co-expressed and co-purified WzyE_PA_-distrepII: His_6_-WzzE_PA_ complex.

The ECA biosynthesis proteins from *P. atrosepticum* were selected for a model system based on expression screening of multiple homologues. Affinity-tagged variants of WzzE_PA_ and WzyE_PA_ were co-expressed in an *E. coli* host, with different combinations of tags used. The predicted complexes were then purified by sequential application to two different affinity columns, with each column specific for one tag. As a result, proteins in the final elution step (E2) must result from a complex possessing both WzzE_PA_ and WzyE_PA_ fusion proteins (figure 1*a*,*b*). To rule out any non-specific effects of the fusion partners, two different affinity tag combinations were used (GST-WzzE_PA_ and WzyE_PA_-His_10_; His_6_-WzzE_PA_ and WzyE_PA_-distrepII). For each tag combination, WzzE_PA_ and WzyE_PA_ co-eluted from the second affinity column (electronic supplementary material, figures S1 and S2). The same outcome was observed with either affinity tag combination, and when the order of the affinity columns was reversed (electronic supplementary material, figures S1 and S2). Control experiments of applying the individual purified proteins to the non-cognate affinity resin showed no significant non-specific binding to the affinity resin (electronic supplementary material, figure S3), demonstrating that non-specific binding does not account for the co-purification.

Analysis of the affinity-purified WzyE_PA_-distrepII : His_6_-WzzE_PA_ complex by blue-native PAGE showed a smeared band and western immunoblots indicated this material contained both proteins (electronic supplementary material, figure S4A). When examined alone by BN-PAGE and western immunoblot, WzyE_PA_-distrepII exhibits a broadly smeared band, with a considerably larger size distribution compared to the sample of the complex. To provide more insight, size exclusion chromatography was performed. This indicated a shift in size of the complex, relative to the individual component proteins (figure 1*c*). When examined alone, WzyE_PA_ and WzzE_PA_ both eluted as broad peaks, whereas the complex eluted as a sharper peak, suggesting conversion to a more homogeneous sample. The elution fraction of the complex was larger than WzyE_PA_ but surprisingly smaller than WzzE_PA_. The latter finding may reflect the complex being more compact than the WzzE_PA_ homo-oligomer. However, an alternative (and perhaps more likely) interpretation is that the average oligomeric state of WzzE_PA_ is constrained upon interaction with WzyE_PA_. This is consistent with the observation of various oligomeric states of isolated Wzz homocomplexes [22–24]. Quantitative immuno-dot blotting was used to estimate the molar ratio of His_6_-WzzE_PA_ : WzyE_PA_-distrepII (electronic supplementary material, figure S4B–D). Known quantities of each protein were spotted in a two-fold serial dilution and the chemiluminescence used to generate a standard curve for each. A serial dilution of the co-purified complex was analysed, and the standard curve used to determine the quantity of each component. This method yielded a stoichiometry of His_6_-WzzE_PA_ : WzyE_PA_-distrepII to be 9.6 (±1.8) : 1.

### 2.2. *In vivo* studies of WzyE/WzzE interaction

**Figure 2.**
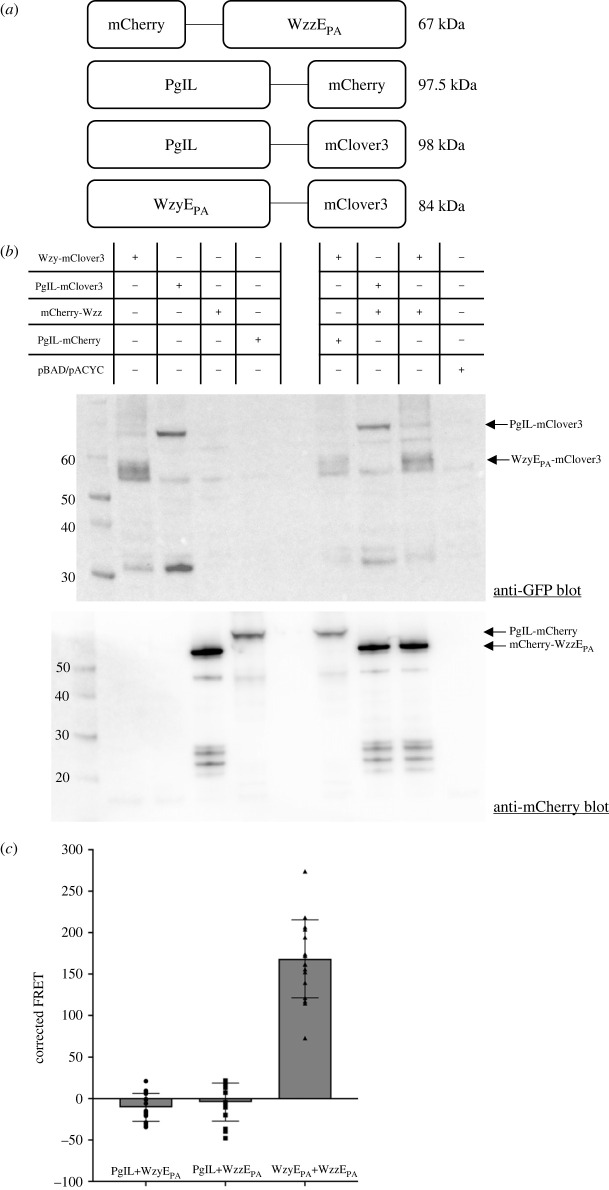
Identification of the *in vivo* WzyE_PA_ : WzzE_PA_ complex by FRET. (*a*) Overview of the domain architecture of the different fluorophore-labelled proteins. (*b*) Various combinations of fluorophore-labelled WzyE_PA_, WzzE_PA_ and control proteins in *E. coli* lysates probed by western immunoblot analysis. (*c*). FRET was measured for the combinations of PglL, WzyE_PA_ and WzzE_PA_ and corrected for background fluorescence and fluorophore bleed-through. *n* = 18; the data represent triplicates and error bars are standard deviation.

To support the isolation of the heterocomplex, the interaction of WzyE_PA_ and WzzE_PA_ was studied *in vivo* in *E. coli* cells. WzyE_PA_ and WzzE_PA_ were fused with fluorophores mClover3 and mCherry, respectively (figure 2*a*), to investigate interactions by Förster resonance energy transfer (FRET). To assess potential non-specific interactions due to the partners being membrane proteins, the PglL oligosaccharyltransferase from *Neisseria meningitidis* [41,42], was expressed as mClover3 and mCherry fusions for negative controls (figure 2*a*). PglL is a membrane protein with multi-spanning membrane topology and, like WzyE_PA_, belongs to the SEDS superfamily but is otherwise unrelated [43]. Each construct was expressed, and western immunoblotting was used to confirm the presence of the desired fusion proteins (figure 2*b*). Fluorescence measurements were conducted on suspensions of *E. coli* cells expressing the different fluorophore-labelled constructs and their combinations. The proportion of baseline fluorescence (i.e. not due to FRET) at 620 nm was measured for each construct by exciting the samples at the mClover3 excitation wavelength (488 nm). The authentic FRET signals were measured for the following pairs of proteins: WzyE_PA_ + WzzE_PA_, WzyE_PA_ + PglL and PglL + WzzE_PA_ (figure 2*c*). A FRET signal was only observed for the WzyE_PA_ : WzzE_PA_ pair indicative that *in vivo* the two proteins form a specific complex *in situ* in the cell, consistent with the data described above for the isolated proteins (figure 2*c*).

### 2.3. High-resolution cryo-EM structure of WzzE_PA_

**Figure 3.**
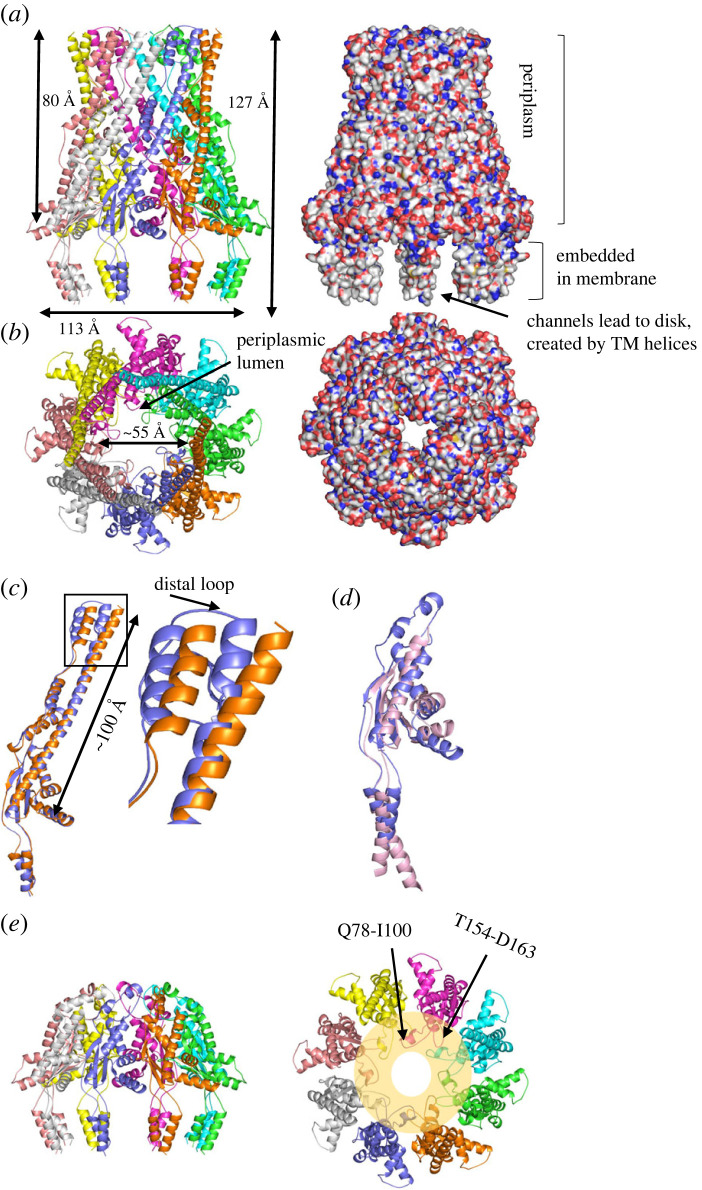
Structure of WzzE_PA_. (*a*) WzzE_PA_ (residue I39–V337) viewed from the side, the protein is represented in cartoon form (left) and in space fill (right). The periplasmic regions enclose a large central lumen. The transmembrane helices create a cavity in the membrane with channels. Residues 1–39 and 338–348 were not visualized. (*b*) WzzE_PA_ viewed from the periplasm looking into the cytoplasm through the protein. The protein is represented in cartoon form (left) and in space fill (right). Space fill shows that the entrance to the lumen from the periplasm is almost occluded. (*c*) The monomers (coloured blue and orange) of WzzE_PA_ are found in two conformations, distinguished by their structures at the periplasmic tip (A222-P280), highlighted in box. The monomers show an RMSD of 1.3 Å over 276 Cα atoms. (*d*) Motif 1 (W58-A204 and R310-E317), the two transmembrane helices (TM1 38–53; TM2 326–337) and the connecting loops are structurally conserved in WzzE_PA_ (blue) and in Wzc_EC_ (pink). (*e*) The side and top view of motif 1 (defined as (*d*)). Motif 1 forms a ring at the periplasmic face of the membrane. Two regions of structure the L-loop (T153-D163) and a loop–helix–loop (Q78-I100) that reach into lumen are highlighted. The L-loop (T153-D163) lies closest to the membrane surface.

A sample of the TEV protease cleaved (removal of affinity tag) and SEC-purified WzyE_PA_ : WzzE_PA_ complex was applied to cryo-EM grids, plunge frozen and reference-free 2D classes were obtained (electronic supplementary material, figure S5A). The classes reveal a detergent belt and the characteristic bell-shaped periplasmic domain of WzzE_PA_ oligomers that has been described previously for oligomers from other species [21–23]. However, no additional density attributable to WzyE_PA_ was evident on the outside or inside of the WzzE_PA_ transmembrane helices. This observation was interpreted as reflecting some combination of complex dissociation during cryo-EM grid preparation, sample heterogeneity and symmetry mismatch. The reconstructed 3D map shows only WzzE_PA_ and that WzzE_PA_ forms an octamer made up of four dimers arranged in C4 symmetry (figure 3*a*,*b*; table 1). By contrast to the recently published full-length structure of *E. coli* K-12 WzzB_EC_ (PDB 6RBG; C8 symmetry), the two transmembrane helices of each monomer (residues 39–54 and 326–337) in WzzE_PA_ were traced and surround a central membrane cavity with a diameter of 57 Å (measured between Ile335 residues, figure 3*a*,*b*). No contacts were formed between the transmembrane helices, although the termini of the transmembrane helices were disordered. The periplasmic domain is dominated by α-helices (figure 3*c*). A prominent conserved feature of the Wzz monomer is a long (approx. 100 Å) helix, which is approximately parallel to the long axis of the molecule [19–23,40]. This helix is connected by a helical turn-containing loop (residues 241–265) to two shorter α-helices, which are arranged approximately anti-parallel to the long helix. In the octamer, there are slight structural differences between the monomers localized at the loop that connects the long helix to the two shorter α-helices and this resulted in C4 symmetry. These helices connect to a region of structure identified as motif 1 [25], which is formed by residues 58–204 and 310–317. In WzzE_PA_, motif 1 consists of a β-sheet with four anti-parallel β-strands and four α-helices (figure 3*d*,*e*). Two regions within motif 1, a loop (residues 153–163) (L-loop) and a loop–helix–loop (78–100), point into the large central periplasmic lumen (figure 3*e*). The L-loop extends further into the lumen and is closer to the membrane than the other region. The interactions between motif 1 of one monomer with its neighbour include salt bridges, hydrogen bonds and van der Waal's interactions, and are dominated by the large helix and the two helices to which it is connected. In the octamer, the periplasmic helices form a barrel that encloses the lumen (figure 3*a*,*b*). The transmembrane helices, which are not closely packed, enclose the disc-shaped cavity within the membrane resembling that seen for other Wzz structures [22,23] (figure 3*a*,*b*). However, comparison with other Wzz structures reveals some structural differences, most notably within motif 1. In the WzzE_PA_ structures, the helix α2 (residues 103–113) connects to α3 (residues 115–124) by a kinked turn of approximately 90°. α3 connects via a loop on the outside of the barrel to α4 (residues 136–149), which connects to the strand β2. This reflects a divergence from the WzzB structures (e.g. WzzB_EC_, RCSB 6RBG [23]), where the α2 helix has no kink and is longer (residue 88–110). The kink in the helix in WzzE_PA_ eliminates regions that participate in inter-subunit contacts in WzzB_EC_. The helix is connected by a seven-residue loop to the strand β2, where the structural elements of WzzE_PA_ and WzzB_EC_ once again superimpose (electronic supplementary material, figure S6). This region of the structure is on the outside of the octamer and some portions make contacts with the neighbouring monomers. Collectively, these changes in structure mean there is very little conservation in the motif 1 contact regions in WzzE_PA_ and WzzB_EC_. Other smaller structural differences occur in the two regions of structures that protrude into the lumen (electronic supplementary material, figure S6).

**Table 1.** Data collection and processing statistics of WzzE_PA_ cryo-EM structure.

*data collection and processing*	
frames	20
total dose (e^−^/Å^2^)	85
pixel size (Å)	1.06
defocus (μm)	1.0–2.7
symmetry	*C*4
movies	2,347
particles	464,832
final number of particles	190,490
refined map resolution FSC = 0.143 (Å)	3.6
map sharpening B-factor (Å^2^)	−151
*refinement*	
model resolution (Å)	2.1/3.7
FSC threshold	0.143/0.5
model resolution range (Å)	1.5–3.7
model composition	
non-hydrogen atoms	18,384
protein residues	2301
*B* factors (Å^2^)	
protein	50
r.m.s. deviations	
bond lengths (Å)	0.004
bond angles (°)	0.790
*validation*	
MolProbity score	1.8
clashscore	11.70
poor rotamers (%)	0.88
Ramachandran plot	
favoured (%)	96.3
allowed (%)	3.3
disallowed (%)	0.4

**Table 2.** List of primers used in this study.

Wzz_fw	AAATTTCCATGGTGAAATCAGAGAACTTG
Wzz_rv	AAATTTCTCGAGACGCAGACGGCGAGCCAG
Wzy_fw	AAATTTATGGCGCTTGGGCAATTTGG
Wzy_rv	AAATTTCTCGAGTTATCTGCTCCTTTATC
gstF1	GGTGATAAAGGAGCAGATACTCGAGGGCGGTGGCGGTTCGTCCCCTATACTAGGTTATTGGAAAATTAAGG
gstR1	CCTGAAAATACAGGTTTTCCTCGAGCGATTTTGGAGGATGGTCGCC
SDM_fw	TTCCCGTCGACAAGCTTGATCCGGCTGCTAACAAAGC
SDM_rv	AAGCTTGTCGACGGGAATTCTTTTTCGAATTGCGGATGACTCC
mClover3_fw	CCGGAA TTCGTGAGCAAGGG
mClover3_rv	CGCAAGCTT CTACTTGTACAGCTCGTC

### 2.4. Detection of WzyE_PA_ in WzyE_PA_ : WzzE_PA_ complexes using an antibody

**Figure 4.**
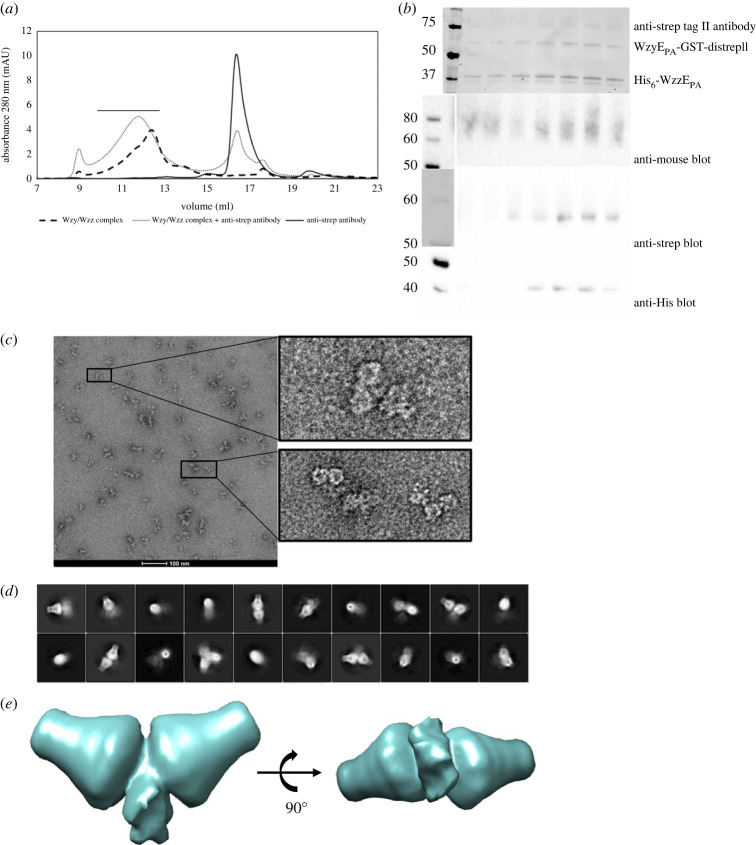
WzyE_PA_ is located within WzzE_PA_. (*a*) Addition of the anti-strep tag II antibody leads to a shift in elution volume of the complex in SEC, as well as to a broadening of the elution peak. Control samples are shown. (*b*) The fractions under the broad peak (indicated by a line above the peak) were analysed by SDS–PAGE and western immunoblot. A polyclonal anti-mouse secondary antibody recognizes the mouse-derived anti-strep tag II antibody bound to WzyE_PA_-GST-distrepII; the HRP-coupled anti-strep tag II antibody was used to detect WzyE_PA_-GST-distrepII and the anti-His antibody was used to probe His_6_-WzzE_PA_. For the western immunoblots, the lanes containing the molecular weight markers have been spliced from the same blot showing the protein lanes in order to align the lanes on the immunoblot containing sample from the same fractions. For the anti-strep immunoblot, a photograph had to be taken of the coloured marker as the antibody did not recognize it. The full-size images of the immunoblots can be found in the electronic supplementary material. (*c*) The concentrated peak fractions were applied to cryo-EM grids and negatively stained with uranyl formate. The inset shows dimeric WzzE_PA_ particles, which were attributed to the antibody cross-linking of WzyE_PA_. (*d*) The reference-free 2D class averages from cryo-EM of the concentrated peak fractions. (*e*) The initial model was generated from 2D class averages obtained from cryo-EM experiments (*d*) showing two WzzE_PA_ molecules adjacent to each other.

To verify the presence and location of WzyE_PA_ in the complex, an antibody-labelling approach was adopted for cryo-EM sample preparation. A monoclonal antibody to distrep tag II (mAB) was added to a sample of affinity-purified His_6_-WzzE_PA_ : WzyE_PA_- GST-distrepII complex, which possesses a WzyE_PA_ fusion protein containing a C-terminal GST and di-strep tag II. Addition of the antibody lead to a shift and broadening of the complex elution peak in SEC (figure 4*a*), whereas incubating the antibody with His_6_-WzzE_PA_ alone did not (electronic supplementary material, figure S7A,B). Samples of the elution peak taken for SDS–PAGE and western blot confirmed the expected presence of all three proteins; WzyE_PA_, WzzE_PA_ and mAB (figure 4*b*; electronic supplementary material, figure S5C–F). Images of negatively stained particles show two flask-shaped structures, characteristic of the WzzE_PA_ oligomeric periplasmic domains, are connected by extra density (figure 4*c*). Single-particle cryo-EM data collected for the WzyE_PA_ : WzzE_PA_ : mAB complexes showed dimerized WzzE_PA_ octamers (figure 4*d*,*e*). A model at approximately 20 Å resolution (electronic supplementary material, figure S7C) was created using these 2D classes, and shows the complex adopts a ‘dumbbell’ arrangement containing the two WzzE_PA_ octamers positioned end to end. The antigen-binding portion of the antibody appears in the centre of the membrane region of the WzzE_PA_ oligomers (figure 4*e*). The location of the antibody indicated that WzyE_PA_ is positioned in the centre of the transmembrane cavity formed by the transmembrane helices of octameric WzzE_PA_. Each half of the dumbbell can accommodate one WzzE_PA_ octamer and the localization of the antibody indicates one WzyE_PA_ monomer is present in the lumen of WzzE_PA_. This result is entirely consistent with the stoichiometry determined by dot blot above.

## 3. Discussion

The polymerization of polysaccharides from lipid-linked repeat units is central to the completion of key bacterial polymers, including ECA, OPS, CPS and peptidoglycan. With the exception of peptidoglycan, these systems exploit a conceptually conserved Wzx/Wzy-dependent pathway, where polymerization is catalysed by Wzy, an integral membrane protein. This biosynthesis strategy is arguably the most abundant format observed in known polysaccharide assembly systems in Gram-positive and Gram-negative bacteria. In addition to Wzy, a member of the PCP family is required to activate and regulate polymerization, resulting in the production of high-molecular weight polysaccharides that sometimes exhibit a modal length distribution, where the chain lengths of most products fall within a relatively narrow range [26,44]. In Gram-negative bacteria, most Wzy-associated PCPs belong to either the PCP-1 (Wzz) or the PCP-2a (Wzc) family [45]. Evidence from genetic data and co-purification methods has accumulated from OPS biosynthesis systems to support the possibility that PCPs form complexes with Wzy and here we extend those observations with ECA biosynthesis. Using FRET labelling and co-purification, we have confirmed that WzzE_PA_ and WzyE_PA_ form a complex *in vivo* (figure 2*c*). Furthermore, using multiple different tandem affinity tagging approaches, we isolated a WzzE_PA_ : WzyE_PA_ complex with a stoichiometry consistent with one WzyE_PA_ protein per WzzE_PA_ octamer (figure 1*d*). Although the proteins are stable and can be purified, we have not confirmed that they are functional in the native organism. The observed stoichiometry is consistent with the lower level of native expression of Wzy in comparison to that of Wzz seen in other bacteria [35,46]. We note that the ECA operon is intact in the *E. coli* C43(DE3) strain used here and we cannot exclude the possibility that WzzE_EC_ monomers are incorporated into (predominantly) WzzE_PA_ octamers to influence stoichiometry determination in the heterocomplex. However, we saw no evidence for significant sequence heterogeneity in the WzzE_PA_ structure that would materially alter our estimate. Consistent with the results reported here, a complex between Wzy_SF_ (*S. flexneri*) and Wzz_pHS-2_ (or Wzz_SF_) has also been observed using a comparable co-purification approach, but the stoichiometry was not established [28].

The 3.6 Å cryo-EM structure of full length WzzE_PA_ that is found as an octamer with a large enclosed central periplasmic lumen connected to a membrane cavity defined by the transmembrane helices (figure 3*a*,*b*, electronic supplementary material, figure S5B). The oligomeric arrangement (figure 3*a*) has been seen in EM structures of Wzz from other species [19,21,23,24] but is different to hexamers/decamers seen in some structures of periplasmic domains [22]. Comparing the WzzE_PA_ and Wzc structures shows that only the transmembrane helices and periplasmic motif 1 (figure 3*d*) are structurally conserved between PCP-1 and PCP-2a proteins (figure 3*d*) [25]. In octamers of both Wzc and WzzE_PA_, the transmembrane helices are arranged in a similar circular array that delimit the cylindrical cavity (approx. 57 Å in diameter), which is embedded within the membrane (WzzE_PA_ shown in figure 3*a*,*b*). The helices are not closely packed and thus create connecting channels between the bulk membrane and the central cavity (figure 3*b*). Motif 1 forms a ring-like structure in the periplasm with two regions extending into the space above the membrane cavity (figure 3*e*). There are several interactions between neighbouring monomers in the octamer that involve residues within motif 1 (figure 3*e*).

**Figure 5.**
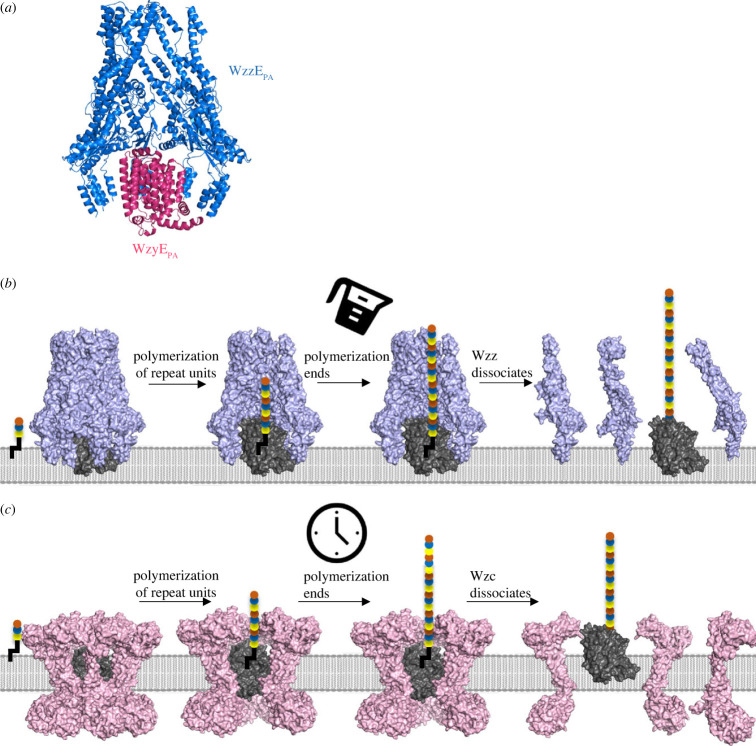
Model for the process of chain length regulation in the Wzx/Wzy pathway. (*a*) AlphaFold model of WzyE_PA_ docked into EM structure of WzzE_PA_. The model of WzyE_PA_ was obtained from AlphaFold (ID: Q6CZF1) and placed by hand into the transmembrane cavity of WzzE. (*b*) The molecular measuring cylinder model of chain regulation of Wzy by Wzz (PCP-1) in ECA and OPS biosynthesis. (*c*) The molecular timer mechanism of chain regulation of Wzy by Wzc (PCP-2a) in CPS biosynthesis.

Using an antibody to a C-terminally tagged WzyE_PA_-distrepII, we visualized the WzzE_PA_ : WzyE_PA_ : mAB complex in both negative stained and cryo-EM (figure 4*c*–*e*). Both methods show that the Fab fragment is centred on the cytoplasmic face of the characteristic Wzz cavity (figure 4*c*,*e*) consistent with the 8 : 1 WzzE_PA_ : WzyE_PA_ *in vitro* stoichiometry (figure 1*d*). The diameter of the membrane embedded portion of the Alphafold model [47,48] of WzyE_PA_, obtained from the AlphaFold Protein Structure Database, is around 50 Å (figure 5*a*), thus the transmembrane portion of WzyE_PA_ can potentially be placed in the WzzE_PA_ (and Wzc) membrane cavity without van der Waal clashes. The surfaces of the transmembrane helices of WzzE_PA_, facing WzyE_PA_, and the membrane-embedded surface of WzyE_PA_ are mainly hydrophobic, consistent with the arrangement deduced from EM (figure 5*a*). AlphaFold-Multimer [49] predictions of a heterodimer consisting of a WzzE chain and WzyE places WzyE in the membrane cavity, in agreement with the EM result.

With WzyE_PA_ placed in the middle of the WzzE_PA_ octamer, a working model for the polymerization process emerges. The gaps between the transmembrane helices of WzzE_PA_ would potentially allow diffusion of the lipid-linked oligosaccharide substrates to access WzyE_PA_, and allow the spent lipid diphosphate carriers to diffuse out (figure 3*a*). In such an arrangement, the periplasmic face of Wzy would interact with the structurally conserved motif 1 (figure 3*e*) that extends into the lumen. Since OPS chain length increases in the presence of Wzz *in vivo* and during *in vitro* synthesis [26], it would suggest that this complex promotes processivity of Wzy and shields the growing polymer from the acceptor (or the action of subsequent enzymes like OPS ligases [3]) until the modal length is achieved. Insertions in motif 1 are known to disrupt the modal chain length regulation of O-antigen [37] consistent with our structural inference that the arrangement of Wzz around Wzy is crucial for function. Wzz mutants with reduced modal chain length are also less able to form stable oligomers [36,37], while Wzz mutants showing increased chain length are all found as oligomers [37]. Further support for this model comes from chimeric proteins composed of fused portions of *S. flexneri* WzzB_SF_ and WzzE_SF_. In the bacterium, these Wzz homologues regulate different Wzy proteins to produce different polymers with different chain length distributions. Exchanging the TM1 of WzzB_SF_ and WzzE_SF_ had little effect. However, creation of a WzzE_SF_ chimera containing TM2 from WzzB_SF_ disrupted ECA modal length regulation, while the reciprocal chimera exhibited a reduced modal length control of the O-antigen [29]. TM1 faces outwards from and TM2 faces inward to the central membrane cavity, and this arrangement aligns with our structural model of WzzE_PA_. Additional studies with *S. flexneri* WzyB_SF_ have identified a region of the protein that is important for its regulation by Wzz_pHS-2_ [28]. This region of WzyB_SF_ was not required for its regulation by Wzz_SF_ [28]. Unfortunately, the primary sequences of WzyE_PA_ and Wzy_SF_ are not well conserved and the AlphaFold models (Wzy_SF_ published in [28]) are sufficiently different that simple comparisons provide limited insight.

The data presented here invalidates one former proposal for Wzz mode of action, in which Wzz serves as a scaffold for multiple Wzy molecules to assemble and where the outer surface of Wzz acts as a ruler [19,22]. The data suggest the nascent polymer forms inside the lumen, consistent with the proposal that the interior surface of the periplasmic domain of WzzE (roughly 80 Å high (figure 3*a*)) may provide some form of a molecular ruler [36]. The interior periplasmic lumen being the key to controlling polymer length was originally suggested in studies with FepE from *E. coli* and WzzB from *S. flexneri*, where mutations within the lumen affected chain length regulation [50,51]. The current data supports a model where the polymer grows until it fills the periplasmic lumen in WzzE. Filling the lumen with polymer may then destabilize the Wzz octamer, which has been shown to exist in different oligomeric states [22–24]. We propose that the putative disruption of the Wzz oligomer serves two purposes. First, since oligomeric Wzz has been shown to be essential for extended polymerization [26,37], disruption of this oligomeric complex would substantially decrease processive polymerization. Second, partial disassembly of Wzz would expose the lipid-linked polymer to the enzymes necessary for transfer to its final carrier or translocation machinery. This explains why mutants lacking PCP function have an ‘unregulated’ phenotype, where product abundance decreases as chain length increases. Once the polymer is transferred into the downstream steps in the pathway, Wzz would be able to re-assemble around Wzy and restart the polymerization cycle (figure 5*b*). This model implicitly introduces a third factor in chain length regulation. In addition to the size of the polymer and volume of the Wzz lumen, the stability of the Wzz oligomer is important. This third factor rationalizes the variation in modal length reported to arise from changes in motif 1 [37]; these would perturb oligomer stability. Although changes to individual residues in motif 1 (e.g. K321T in *Pseudomonas aeruginosa* Wzz2 [36]) have resulted in shifted modal length, mechanistic interpretation has not been possible. A comprehensive study in which a pentapeptide was inserted into different regions of Wzz_SF_ showed that changes in most regions resulted in proteins conferring decreased modal length, and some completely eliminated regulation [37]. Like the site-directed mutants, many of the phenotypes of insertions can be rationalized by invoking a decrease in oligomer stability. Interestingly, the L-loop of Wzz (electronic supplementary material, figure S6) is predicted to engage Wzy and insertions in this loop led to an increase in modal length [37].

Conceptually the model has parallels with the CPS system involving Wzc (PCP-2a), where polymerization of CPS requires both assembly and disassembly of the Wzc octamer; in the case of Wzc, the disassembly process is driven by phosphorylation [25]. AlphaFold models of the relevant Wzy show that the majority of the protein would be embedded within the membrane. This would suggest that the two TM helices and motif 1 of Wzz (and by analogy Wzc), which are located in the membrane and immediately above the location the Wzy polymerase would occupy, are central to the interaction between polymerase and co-polymerase. Consistent with this concept, we note that is only this region that is structurally conserved in Wzz and Wzc. We propose these regions engage and modulate Wzy. Modulation of Wzy would be achieved by filling the lumen in PCP-1 systems (akin to a molecular measuring cylinder) (figure 5*b*), while PCP-2a systems depend on the kinetics of the competing phosphorylation/dephosphorylation to exercise control (analogous to a molecular timer) (figure 5*c*).

Mutant complementation experiments with *wzx*, *wzy* and *wzz* knockout strains have led to the proposal that Wzx (which flips the Wzy substrate across the membrane), Wzy and Wzz form a multi-protein complex [52,53]. A super-complex of this type is enticing because it forms a central coordinating point to regulate OPS biosynthesis by interacting with other proteins and can be extended to the biosynthesis of other types of polysaccharides assembled by the same strategy. A wider network of regulation would be consistent with the observation that deletions in the proposed periplasmic loop between TMHs 4 and 5 in WbaP (the phosphoglycosyltransferase initiating enzyme for OPS synthesis in *Salmonella*) affect OPS chain length while retaining catalytic activity [53]. It has been hypothesized that this deletion affects the interaction with the chain length regulator. Experimental evidence for this complex is currently lacking.

In conclusion, we have reported data consistent with new mechanistic model for control of Wzy-mediated polymerization by the PCP-1 proteins. A fuller molecular understanding of this and other PCP-regulated systems will require an atomic level resolution of the interactions between Wzy, its regulators, and other participants in a super-complex. This is the long-term goal of our research.

## 4. Methods

### 4.1. Cloning

The genes encoding WzzEPA (Uniprot ID: Q6CZE3; gene locus ECA4209) and WzyEPA (UniProt ID: Q6CZF1; gene locus ECA4201)) were PCR amplified from genomic DNA of *P. atrosepticum* SCRI 1043 (ATCC BAA-672). The DNA fragment encoding WzzE_PA_ was cloned into pACYCGSTDuet (Novagen) and pEHisTEV [54] adding an N-terminal GST-tag or a TEV protease-cleavable His_6_-tag, respectively, to the gene product. The DNA fragment encoding WzyE_PA_ was cloned into a proprietary vector constructed by Huanting Liu, or into pBAD10HisTEV, adding a C-terminal TEV protease-cleavable distrepII tag or His_10_-tag, respectively. All constructs were cloned using NcoI and XhoI restriction sites introduced by PCR primers. For the WzyE_PA_-GST-distrepII construct, *E. coli* GST was inserted between Wzy and the C-terminal distrep tag II. The GST-encoding fragment was amplified from plasmid pGEX-KT by PCR using KOD DNA polymerase. pGEX-KT was obtained from the American Type Culture Collection (ATCC77331). The vector containing the *wzy*E_PA_-distrepII fragment was linearized with XhoI and the GST-encoding fragment was inserted at this site by Gibson Assembly (New England Biolabs). For the Wzy-distrepII-mClover3 construct, the *mClover3* gene was cloned by PCR amplification from a plasmid purchased from Addgene (ID: 105802); EcoRI and HindIII restriction sites were in the primer sequences, and the amplification product was cloned into the pBAD10HisTEV vector containing Wzy-distrepII at the 3′ end of the fragment encoding the di-strepII tag. The vector was modified by site-directed mutagenesis to incorporate the EcoRI and HindIII restriction sites [55]. The same vector was used to express PglL-distrepII-mClover3. The *mCherry* fragment was PCR amplified, introducing BamHI and EcoRI restriction sites, and the amplification product was cloned into the pACYCDuet-1 vector. Subsequently, *wzz*E_PA_ was cloned into the same vector at the 3′ end of *mCherry* by PCR amplification and restriction digest using EcoRI and XhoI. *PglL* was cloned into the *mCherry* containing pACYCDuet-1 vector by PCR amplification and restriction digestion using NcoI and BamHI, introducing *pglL* at the 5′ end of *mCherry*. The primers used are listed in table 2.

### 4.2. Expression and purification

Chemically competent *E. coli* C43(DE3) F^–^ ompT gal dcm hsdSB(r_B_^−^ m_B_^−^) cells [56] were transformed sequentially by the two plasmids [57]. The culture volume for individual protein expression was 6 l, whereas the complexes of different affinity-tagged WzyE_PA_ and WzzE_PA_ were cultured in 12 l Luria Broth. WzyE_PA_-His_10_ was co-expressed with GST-WzzE_PA_ at 25°C for 4 h following induction of expression with 0.1 mM IPTG and 0.2% (w/v) arabinose at OD_600_ 1.0. WzyE_PA_-distrepII was co-expressed with His_6_-WzzE_PA_ at 15°C overnight, following induction with 0.5 mM IPTG at OD_600_ 0.4. Cells were harvested by centrifugation at 5000 g, 4°C and cell membranes were prepared by lysing the cells in PBS, pH 7.2 using a cell disrupter, removing cell debris at 15 000 × g, and ultracentrifugation at 150 000 × g. Membranes were solubilized in PBS containing 1% (w/v) DDM for 2 h at 4°C. The supernatant containing solubilized proteins was obtained by centrifugation at 150 000 × g.

All affinity chromatography steps were done as batch purifications at 4°C. For the purification with the GST-tag, proteins were bound to glutathione sepharose 4B resin (Cytiva), washed in PBS containing DDM, and eluted in 50 mM Tris pH 8.0, 200 mM NaCl, 10 mM glutathione, 0.026% (w/v) DDM. The eluate was desalted into PBS, pH 8, 20 mM imidazole, 0.026% (w/v) DDM and bound onto HIS-Select cobalt resin (Sigma Aldrich). After washing with PBS, pH 8, 30 mM imidazole, 0.026% (w/v) DDM, the protein was eluted in PBS, pH 8, 400 mM imidazole, 0.026% (w/v) DDM. For the reverse purification, protein was bound to nickel resin (GE Healthcare), washed with PBS containing 20 and then 30 mM imidazole before being eluted using 400 mM imidazole. The eluate was dialyzed into PBS, pH 8.0, 0.026% (w/v) DDM and bound to glutathione sepharose 4B. The resin was washed with PBS, pH 8.0, 0.026% (w/v) DDM and bound protein was eluted using 50 mM Tris pH 8.0, 200 mM NaCl, 10 mM glutathione, 0.026% (w/v) DDM.

For the purification of His_6_-WzzE_PA_ and WzyE_PA_-distrepII, the supernatant was incubated with either HIS-Select cobalt resin (Sigma Aldrich) or Talon resin (Cytiva). The resin was washed with PBS, pH 7.3, 20 mM imidazole, 0.026% (w/v) DDM and bound proteins eluted with 400 mM imidazole. The eluate was dialyzed in 50 mM phosphate buffer, pH 8, 200 mM NaCl, 0.026% (w/v) DDM overnight and then incubated with streptactin or streptactinXT resin (IBA Lifesciences). The resin was washed with dialysis buffer and bound protein eluted in buffer containing 2.5 mM desthiobiotin.

The elution fraction was concentrated in a spin concentrator and either directly injected onto a superose 6 10/300, or incubated with TEV protease for 3 h at 4°C, prior to size exclusion chromatography.

### 4.3. SDS–PAGE analysis

Samples for SDS–PAGE analysis of the purification progress were prepared using 2× Laemmli sample buffer (Bio-Rad). Samples for analysis via SDS–PAGE followed by western immunoblotting were loaded onto a 4–12% Bis–Tris NuPAGE gel (Novex), along with Sharp Pre-stained protein standard (Novex) and run at 200V in 1× MES SDS running buffer (NuPAGE). Samples for SDS–PAGE analysis were loaded onto an any-kD Mini Protean TGX Stain-free protein gel (Bio-Rad), along with Precision plus unstained marker (Bio-Rad) and run at 300 V in 1× Tris/Glycine/SDS buffer (Bio-Rad). Any-kD Mini Protean TGX Stain-free protein gels were visualized using the Chemidoc MP system (Bio-Rad). The loaded sample volume ranged between 15 and 20 µl.

### 4.4. Blue-native PAGE and western immunoblot

For blue-native PAGE, samples were prepared by mixing with NativePage sample buffer and NativePage 5% G-250 Sample Additive (both Thermo Fisher Scientific) and separated by electrophoresis on a NativePAGE Novex Bis–Tris 4–16% precast gel (Thermo Fisher Scientific). NativeMark Unstained Protein Standard was used for reference (Thermo Fisher Scientific). For western immunoblotting, the PVDF membrane was air-dried after transfer, rinsed in methanol and water, blocked with 5% milk in PBS-T (PBS with 0.05% (v/v) Tween-20). For detection, the immunoblot was probed with HRP-conjugated anti-His tag (Sigma Aldrich) and anti-strep tag II (IBA Lifesciences) antibodies.

### 4.5. Dot blot to determine stoichiometry in protein complexes

Samples were applied directly onto a nitrocellulose membrane using the Minifold 96-well dot-blot system (GE Healthcare). To establish a standard curve, three different amounts of co-purified complex were applied, along with a serial dilution of His_6_-WzzE_PA_ and WzyE_PA_-distrepII. After membrane blocking with 5% milk in PBS-T, the membrane was probed with HRP-coupled anti-His tag (Sigma) or anti-strep tag II (IBA Lifesciences) antibodies. The signals were quantified using the BioRad ChemiDoc MP system with background correction. The circles on the dot blot indicate the areas used for densitometry. U1 indicates the area used for background correction. Wzz exhibited a double band on SDS–PAGE, only the top band could be detected by the anti-His antibody during western blotting, whereas the second lower band did not. To account for this untagged Wzz in the sample, the ratio between tagged/untagged protein was determined from an SDS–PAGE gel using the BioRad ChemiDoc MP system.

### 4.6. Sample preparation for electron microscopy

For the high-resolution data collection for WzzE_PA_, tandem affinity-purified and TEV-cleaved WzzE_PA_ : WzyE_PA_ was subjected to SEC on a superose 6 column and the peak fractions containing purified complex were pooled and concentrated to 5 mg ml^−1^. To ensure that the complex was present in ice suspended over holes, the sample was applied to grids that had been glow-discharged in the presence of pentyl amine and then plunge frozen on a Vitrobot mark IV (Thermo Fisher Scientific). Grids were imaged on a Thermo Fisher Scientific Titan Krios equipped with a Gatan BioQuantum energy filter and K2 camera. Data were recorded at a nominal magnification of 130 k×, corresponding to 1.06 Å pixel^−1^ at the specimen scale. The total electron dose was 85 e^−^/Å^2^, partitioned over 20 movie frames, with defocus ranging between −1 and −2.7 µm; 2347 micrographs were recorded and processed to correct for specimen movement in Relion 3.1 [58]. Defocus estimation was performed using CtfFind4 [59]. From these data, a total of 464 832 putative particle images were picked for further processing in Relion 3.1 [58]. 2D and 3D classification defined a final dataset of 190 490 particle images that were refined to calculate a final reconstruction with a resolution estimate of 3.6 Å. To aid the interpretation of density in less well resolved areas of the map, the reconstruction was subject to deep-learning based density modification using DeepEMhancer [60]. Model building was performed using Coot [61] and Phenix [62–64].

For negative staining of His_6_-WzzE_PA_ : WzyE_PA_-GST-distrepII complexes with anti-strep tag II antibody (mAB), the elution from the streptactin resin (IBA Lifesciences) (or in case of His_6_-WzzE_PA_, the elution from the cobalt resin), was concentrated in a spin concentrator and incubated with the anti-strep tag II antibody overnight at 4°C. The mixture was injected onto a superose 6 10/300 (Cytiva) column. A sample of the peak fraction was applied on CU400 grids and stained with 2% uranyl acetate. The grids were imaged on a FEI Tecnai T12 microscope.

For single-particle cryo-EM of WzyE_PA_-distrepII : His_6_-WzzE_PA_ in complex with the IgG-class anti-strep tag II antibody, fractions containing the proteins of interest were pooled and concentrated. Sample was applied to glow-discharged Quantifoil 200 Au mesh R1.2/1.3 grids and plunge frozen using a Vitrobot Mark IV. 1621 micrographs were collected on a FEI Glacios microscope equipped with a Falcon III camera in linear integrating mode. Processing was conducted in Relion 3.1 [57,58] and motion correction was performed using MotionCor2 [64,65]. Ctf estimation was done using Gctf [65] and particles were manually picked to create a template. The template was used for auto-picking in Relion 3.1, and 15 442 particles were picked and used to generate an initial model without symmetry application. Based on the observed symmetry of the obtained model, the same particles were used for initial model generation with C2 symmetry applied followed by a round of 3D refinement.

### 4.7. *In vivo* Förster resonance energy transfer

The individual vectors as well as plasmids expressing the various combinations of WzyE_PA_-mClover3, mCherry-WzzE_PA_ and the PglL fusion proteins, PglL-mClover3 and PglL-mCherry were transformed into *E. coli* C43(DE3) cells. The combination of the empty pBAD and pACYCDuet-1 vectors were also transformed. Single colonies of each transformant were selected and grown in LB media in the presence of antibiotics and 1% (w/v) d-glucose overnight at 37°C. The culture was diluted 25-fold into LB with appropriate antibiotics and grown at 37°C to OD 1.2–1.3. Cloned gene expression was induced with 0.002% (w/v) l-arabinose, 0.4 mM IPTG or both and the cells were grown overnight at 15°C. Cells were harvested by centrifugation, washed in PBS, diluted 2-fold in PBS and cell density was measured at 450 nm and used for OD correction to 1. Fluorescence was measured in a Clariostar plate reader (BMG Labtech) at 488–14 nm/535–30 nm for mClover3 and at 570–15 nm/620–20 nm for mCherry. FRET measurements were conducted at 488–14/620–20 nm. Cell density was measured at 450 nm and used for OD correction to 1. To obtain the corrected FRET value, measurements were corrected for background using the empty vector control and for baseline fluorescence by measuring the single vector control samples [65]. Measurements were conducted in triplicate. Data were plotted in GraphPad Prism 9 as a scatter plot with bar diagram.

To confirm expression of the fusion proteins, samples of the cells from the FRET experiments were taken for western immunoblot analysis using the HRP-conjugated anti-GFP-tag monoclonal antibody and the polyclonal anti-mCherry antibody (Proteintech).

## Data Availability

EM maps and models are deposited in the EMDB and wwPDB under accession codes EMD-16364 and PDB 8C0E. Raw data are deposited in EMPIAR under accession code EMPIAR-11355. All plasmids are being deposited with ADDGENE, until their release they are available as *E. coli* agar stabs upon request to J.H.N. The data are provided in electronic supplementary material [66].
